# Total Ionizing Dose Effect Simulation Modeling and Analysis for a DCAP Power Chip

**DOI:** 10.3390/mi16080917

**Published:** 2025-08-08

**Authors:** Xinfang Liao, Danyang Lei, Yanjun Fu, Yuchen Liu, Kangqi Huang, Yuan Wei, Yinghong Zuo, Yashi Ying, Yi Liu, Changqing Xu, Yintang Yang

**Affiliations:** 1 Guangzhou Institute of Technology, Xidian University, Guangzhou 510555, China; xinfangliao_xidian@163.com (X.L.); ldy07294553@163.com (D.L.); yingyashi2000@163.com (Y.Y.); 2 Northwest Institute of Nuclear Technology, Xi’an 710024, China; fuyanjun@nint.ac.cn (Y.F.); weiyuana@nint.ac.cn (Y.W.); zuoyinghong@nint.ac.cn (Y.Z.); 3 School of Microelectronics, Xidian University, Xi’an 710071, China; 23211215184@stu.xidian.edu.cn (Y.L.); kasch_h@163.com (K.H.); ytyang@xidian.edu.cn (Y.Y.)

**Keywords:** total ionizing dose effect, power chip, fault injection model, radiation sensitivity analysis

## Abstract

In this paper, a systematic study on performance degradation of a 0.18 μm BCD-process DCAP (Direct connection to the output CAPacitor) power chip under a total-dose radiation environment is carried out. The effects of total-dose radiation on the electrical characteristics of an MOS device are analyzed through device-level simulation. Based on the simulation results, a total-dose fault injection model is established and applied to a circuit-level simulation of the DCAP power chip. Our simulation modeling and analysis results show that total-dose radiation degrades output voltage accuracy and switching frequency, to which the bandgap reference circuit is identified as the most sensitive module. The findings presented in this paper provide theoretical support for total-dose radiation hardening designs for the DCAP power chip.

## 1. Introduction

With the development of space technology, electronic systems are increasingly used in space exploration, satellite communications and deep-space exploration. The normal operation of these systems depends on efficient and stable power chips [1]. In order to meet different functional module requirements, power chips need to convert input power into multiple voltage levels [2]. However, radiation sources such as energetic particles, cosmic rays and nuclear explosions in the space radiation environment pose threats to power chips. Among the effect of space radiation, the total ionizing dose (TID) effect has been shown to be a major cause of radiation damage. It can lead to accumulation of charge in the oxide layer in MOS devices, and also to the generation of interfacial defects [3,4,5], resulting in performance degradation and even failure of the power chip [6].

The power chip is the core of an electronic system, and its reliability directly affects overall system function. According to statistics from NASA (National Aeronautics and Space Administration), 20% to 30% of spacecraft failures originate from the power chip [7], and its reliability in a radiation environment is particularly important. Under extreme conditions such as a nuclear explosion in space or a solar energetic particle event, the total ionizing dose effect may lead to phenomena such as output voltage drifts and switching-frequency abnormalities in power chips, which may in turn affect the stability of electronic systems, jeopardizing mission reliability [8,9].

Therefore, studies involving total ionizing dose effect modeling of key devices and circuit-level simulation in power chips are crucial, as they help to assess performance degradation, reveal failure mechanisms and guide radiation hardening designs for application in the space radiation environment. In this paper, using a 0.18 μm DCAP-type power chip, we establish a fault injection model for an MOS device based on device-level total- dose simulation results. Then, in combination with circuit-level simulation, we carry out a complete total-dose performance damage analysis for the DCAP power chip. The research in this paper provides theoretical support for radiation hardening designs for DCAP power chips [10,11,12,13,14,15].

## 2. Device-Level Simulation and Modeling

### 2.1. MOS Device Total Ionizing Dose Effect Simulation

According to experimental and simulation studies in the existing literature, under the total ionizing dose radiation condition, PMOS devices are relatively unaffected, compared with NMOS devices [16,17,18]. In this study, therefore, we focused on an NMOS device for analysis of radiation effects.

**Figure 1:**
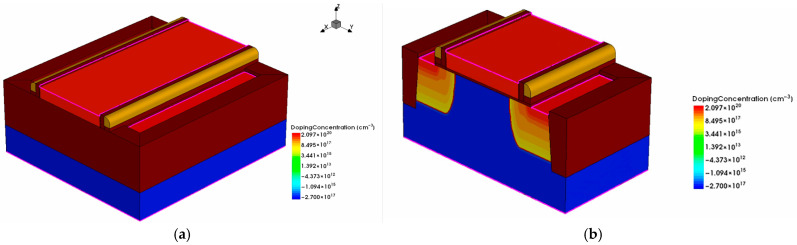
(**a**) 3D view of the 5 V NMOS device. (**b**) Schematic diagram of the cross-section along the device channel.

**Figure 2:**
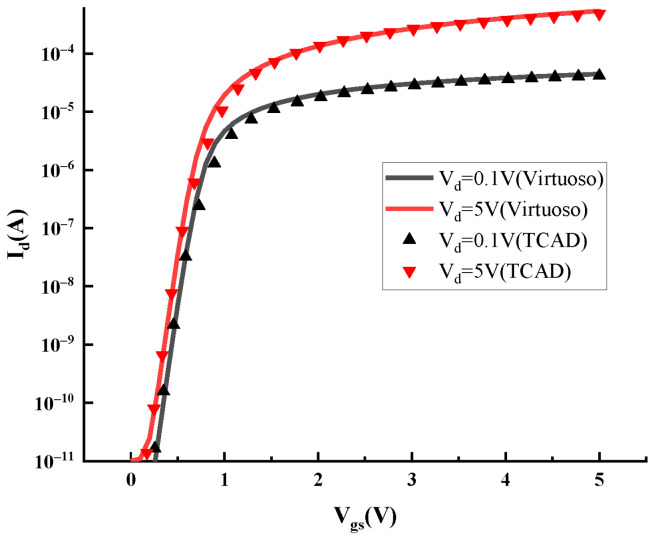
Comparison of transfer characteristic curves between the TCAD simulation model and SPICE model.

For a 5 V NMOS device which is mainly used in the 0.18 μm BCD-process DCAP power chip, we employed Sentaurus TCAD software (version 2018.06-SP2) to simulate its total-dose radiation effect. The constructed device model is shown in Figure 1. In order to validate the accuracy of the TCAD simulation model, we also constructed a corresponding SPICE model for the 5 V NMOS device using Cadence Virtuoso software (version IC6.1.8), and we obtained its transfer characteristics at V_d_ = 0.1 V and V_d_ = 5 V for reference characteristic curves. The TCAD simulation model was calibrated by adjusting and optimizing the structure parameters of the 5 V NMOS device to match the reference characteristic curves. The successfully calibrated transfer characteristic curves are shown in Figure 2.

In an MOS device, the total ionizing dose effect affects the device characteristics through charge accumulation within the oxide layer and the generation of trap charge at the SiO_2_-Si interface [19,20]. In this paper, the Insulator Fixed Charges model in the Sentaurus TCAD software was used to simulate the radiation-induced trap charge and its distribution. This enabled the influence of the total ionizing dose effect on the electrical characteristics of the 5 V NMOS device to be observed.

The gate oxide thickness of the 5 V NMOS device in the DCAP power chip studied in this paper was 13.3 nm. Experimental studies of similar processes and similar gate oxide thicknesses [21,22] have shown that the leakage current caused by the gate oxide is negligible, and that the maximum drift quantity of the threshold voltage under an irradiation dose of 500 krad is 0.0373 V, a level which has a very limited effect on the device characteristics [23]. In this paper, therefore, the total ionizing dose effect simulation of the 5 V NMOS device was focused on modeling and analyzing the trap charge at the STI field oxide layer and its interface.

**Figure 3:**
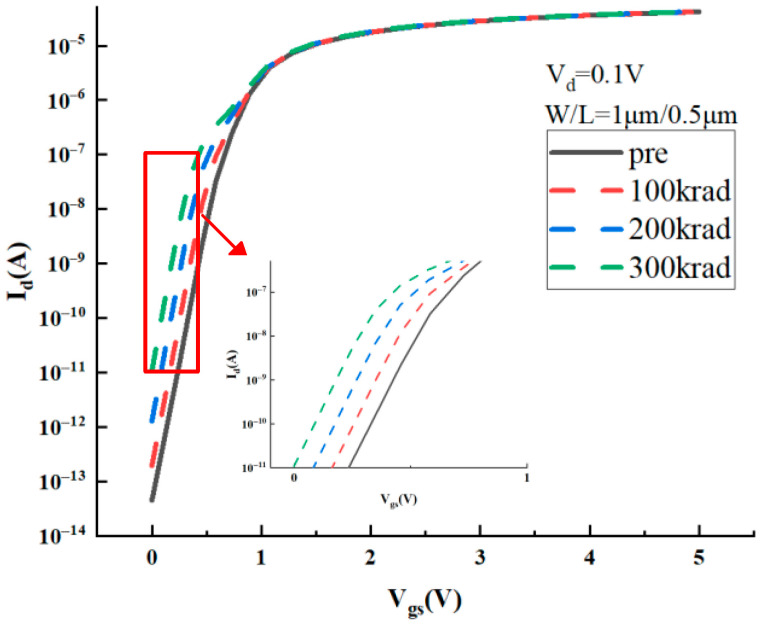
I_d_-V_gs_ curves for the 5 V NMOS device before and after radiation under different doses.

Figure 3 shows the I_d_-V_gs_ curves for the 5 V NMOS device with a width-to-length ratio W/L = 1 μm/0.5 μm at a drain-source voltage of 0.1 V before and after radiation. The solid line indicates the pre-radiation curve, and the dashed lines indicate the characteristic curves for different cumulative doses. The simulation results show a significant increment in the leakage current under the total-dose radiation, consistent with the research data in [24,25].

### 2.2. MOS Device Fault Injection Modeling

**Figure 4:**
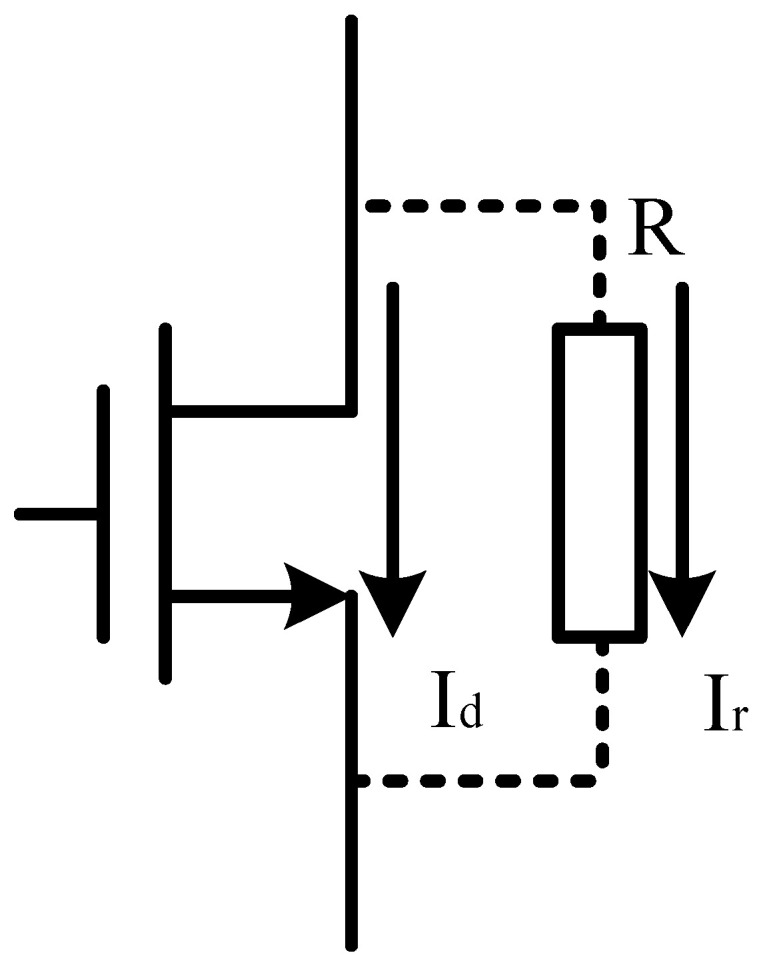
Fault injection model of the 5 V NMOS device.

For research on circuit-level total ionizing dose effect simulation, a fault injection model must be introduced to model the increased leakage current in the irradiated device, which can generally be described as a controlled current source or a resistor in parallel [26]. However, because the leakage current is small and the magnitude of the leakage current is close to that of the bias current near the threshold voltage, the controlled current source may lead to a large deviation in the simulation results. In addition, in SPICE simulation of complex circuits, the addition of nonlinear current sources results in significant increases in simulation time and resource consumption, and the simulation is also more prone to non-convergence. Therefore, in the present study of the DCAP power chip with hundreds of MOS devices, in order to ensure simulation precision and convergence, the total ionizing dose effect was modeled by a resistor in parallel between the drain and source of the 5 V NMOS device [27]. Figure 4 shows the schematic diagram of the fault injection model.

Because the device leakage current varies with the voltage between the drain electrode and the source electrode, it is difficult to accurately model the total ionizing dose effect of a DCAP power chip using a fixed resistor. Therefore, based on the device-level simulation results, the drain-source voltage was sampled at small steps from 0 V to 5 V when the gate-source voltage was 0 V, and the resistor value was calculated through Ohm’s law to generate a nonlinear lookup table in which different irradiation doses corresponded to different resistance values. Then, the data was processed by the perl script to generate the corresponding Verilog-A code, and the establishment of the fault injection model for the circuit-level total ionizing dose effect simulation was completed. The model could therefore consider the effects of both the drain-source voltage and irradiation dose.

**Figure 5:**
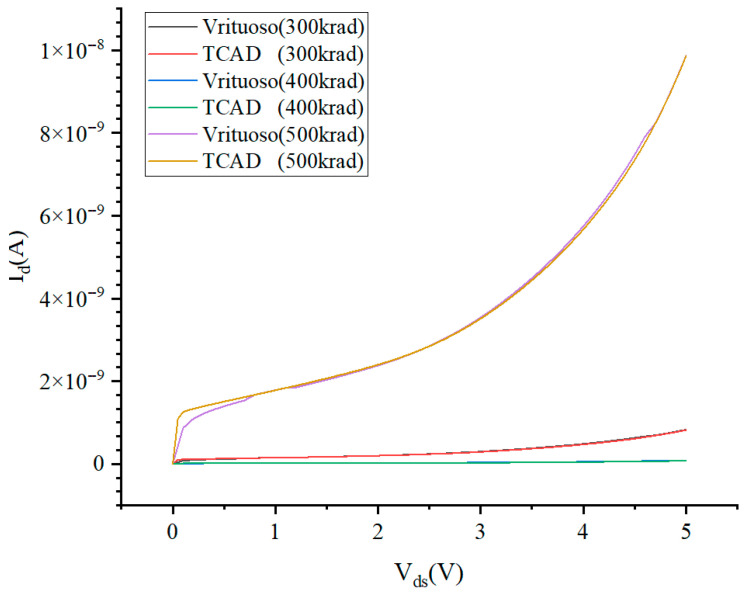
I_d_-V_ds_ curves at different irradiation doses for circuit-level and device-level simulations.

To verify the accuracy of the fault injection model, a total ionizing dose effect simulation circuit for the 5 V NMOS device was constructed using the Cadence Virtuoso software. The BSIM4 model was used to describe the intrinsic characteristics of the non-irradiated device, and the fault injection model was added to the drain-source terminals to analyze the total ionizing dose effect. Figure 5 shows the output characteristic curves generated by the circuit-level simulation and the device-level simulation at different irradiation doses, from which we can observe that the curves are well fitted, verifying the accuracy of the fault injection model for the 5 V NMOS device.

## 3. DCAP Power Chip Simulation and Discussion

**Figure 6:**
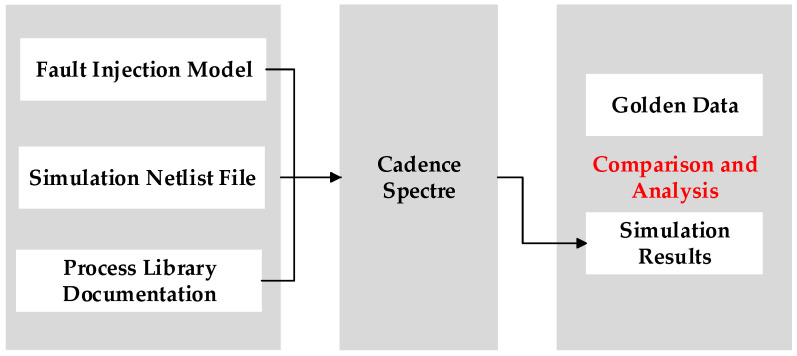
Flow chart for total ionizing dose effect simulation.

The total ionizing dose effect simulation for the DCAP power chip is shown in the form of a flow chart in Figure 6. The flow may be expressed as follows: First, select the corresponding fault injection model according to the simulation requirements, combined with the simulation netlist and process library. Then, inject the faults at the sensitive devices through the script. On this basis, generate the radiation model netlist and run the Spectre simulation. Finally, compare the simulation results with the golden data without radiation to evaluate the impact of total ionizing dose effect on circuit performance.

### 3.1. System Performance Simulation

**Figure 7:**
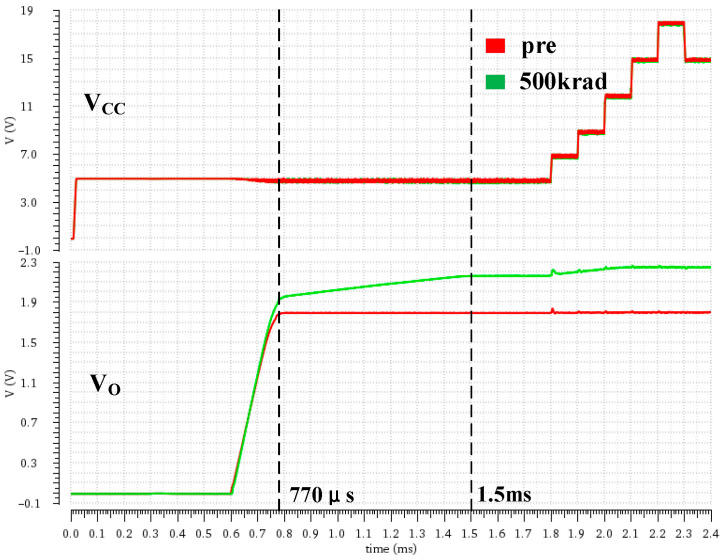
Input and output voltages before and after total-dose radiation (input voltage 5 V to 18 V).

According to the DCAP power chip design indicators, the output voltage ripple and accuracy were simulated and analyzed under the total-dose radiation environment. The simulation was carried out under a typical process condition (TT Corner). The output voltage was set to 1.8 V through a fixed feedback resistor. The input was controlled by a voltage source, and the input curve was consistent before and after radiation. Figure 7 illustrates the simulation results, which show that the output voltage builds up gradually after the chip starts up for about 0.6 ms. Under an irradiation dose of 500 krad, the output voltage rises significantly and the stabilization time extends from 770 μs to about 1.5 ms, during which time there is a slow increase in the process, indicating a reduction in steady-state accuracy and a slower response caused by the total ionizing dose effect.

**Figure 8:**
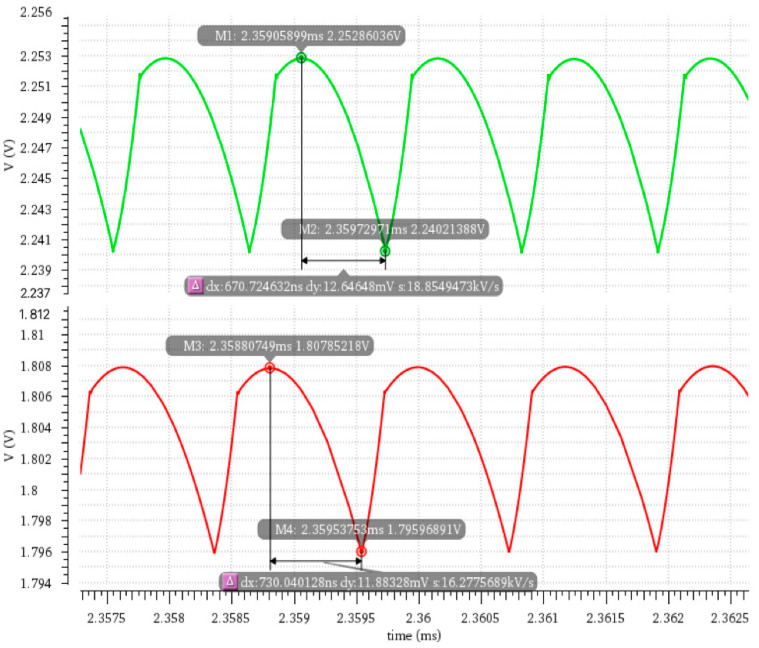
Output voltage ripple characteristics before and after total-dose radiation (input voltage 15 V). The green line represents the curve after radiation, and the red line represents the curve before radiation.

Figure 8 shows a comparison of the output voltage ripple for the DCAP power chip before and after total-dose radiation at an irradiation dose of 500 krad, where the upper part is the curve after radiation, and the lower part is the curve before radiation. In addition, according to the corresponding simulation data, Table 1 calculates and shows the amplitude and accuracy of output voltage ripple before and after radiation as a function of the input voltage. It can be observed from Table 1 that, for the DCAP power chip without radiation, output accuracy is controlled within ±0.15% and ripple amplitude is controlled within ±6.5 mV when the output voltage is 1.8 V and the load current is 3 A, indicating that chip performance is stable. It should be pointed out here that output accuracy is typically measured by the deviation range of the output voltage relative to the theoretical value. However, for the DCAP power chip after radiation, output accuracy decreases significantly, with a maximum deviation of 24.92% and a minimum deviation of 20.18%, far exceeding the conventional design indicator. This reflects the serious impact of the total ionizing dose effect on output voltage stability. In contrast, the output voltage ripple amplitude slightly increases to ±7 mV, and the variation is not significant. Meanwhile, the line regulation, which is used to describe the change in the output voltage resulting from a specified change in the input voltage, is calculated to increase from 0.26% to 3.57% after radiation, further verifying that the circuit’s output voltage stability decreases dramatically when the input voltage fluctuates under the total-dose radiation environment.

**Figure 9:**
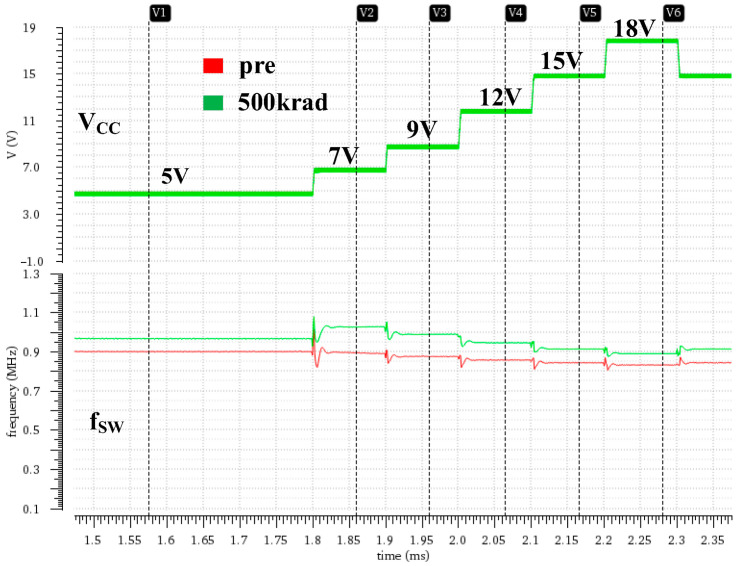
Variation curves for switching frequency versus input voltage before and after total-dose radiation.

Figure 9 shows curves for switching frequency versus input voltage before and after total-dose radiation, and Table 2 presents the corresponding data. The simulation results demonstrate that, under the total ionizing dose effect, the switching frequency increases and the frequency fluctuation range increases with input voltage from ±7.4% to ±13.1%, indicating that the constant frequency characteristics of the DCAP architecture are significantly degraded. In combination with the circuit structure analysis, the switching frequency of the system is controlled by the adaptive constant on-time module, which delivers frequency stabilization by dynamically adjusting the on-time. In addition, an increase in the switching frequency may result from an increase in the output voltage. To maintain the regulated output, the system automatically increases the switching frequency to compensate for an increase in the output voltage.

### 3.2. Sensitivity Analysis for Key Modules

In order to identify the module most sensitive to the total ionizing dose effect in the DCAP power chip, and to provide theoretical support for the radiation hardening design, a single-point fault injection method was adopted for each module studied, so that we could keep the remaining modules free from radiation and assess the impact of the total ionizing dose effect for each module on system output accuracy and stability. For the DCAP power chip researched in this paper, the bandgap reference module, adaptive constant on-time module and ripple compensation module carry out the core functions of reference signal generation, switching frequency control and ripple suppression, respectively, corresponding to different performance parameters of the DCAP power chip. Therefore, these three key modules were selected for the single-point fault injection simulation so that their respective sensitivities to the total ionizing dose effect could be revealed.

**Figure 10:**
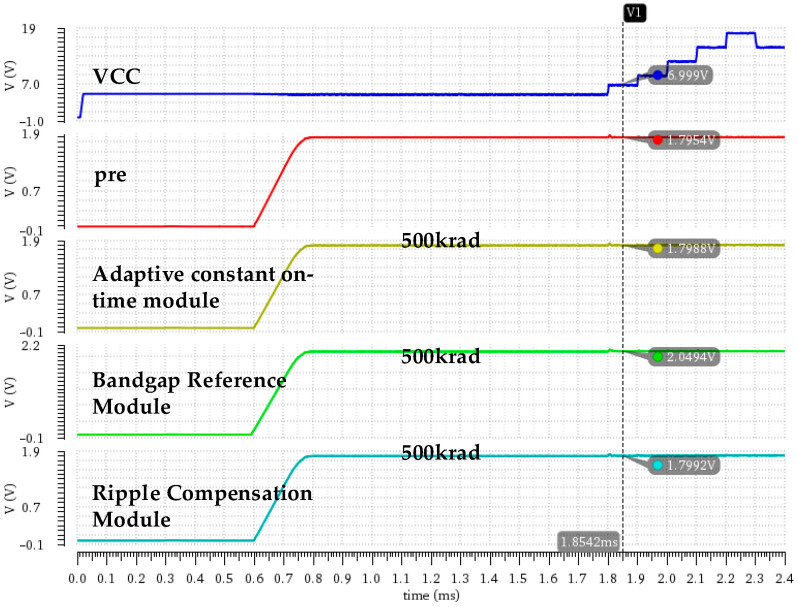
Variations in output voltage after introducing the total-dose fault injection model into each key module.

Figure 10 illustrates variations in output voltage after irradiating each key module with the total-dose fault injection model. The simulation results show that the output voltage is basically smooth after the circuit is stabilized, but there are significant differences in the voltage drifts caused by different modules. Table 3 shows the detailed simulation data, from which we may determine that, under the total-dose radiation, the bandgap reference module has the most significant impact on output accuracy. For example, under an irradiation dose of 500 krad and an input voltage of 5 V, the output voltage deviation caused by the bandgap reference module is as high as +13.18%, significantly higher than the deviations caused by other modules. The reason is that the bandgap reference module is used to provide a stable reference voltage for the system; therefore, degradation of the bandgap reference module directly causes a synchronous increase in the output voltage, leading to a significant decrease in accuracy. In contrast, the adaptive constant on-time module and ripple compensation module mainly affect the switching frequency and output voltage ripple, so they have less impact on output accuracy, which can be seen to exhibit only a slight degradation.

**Figure 11:**
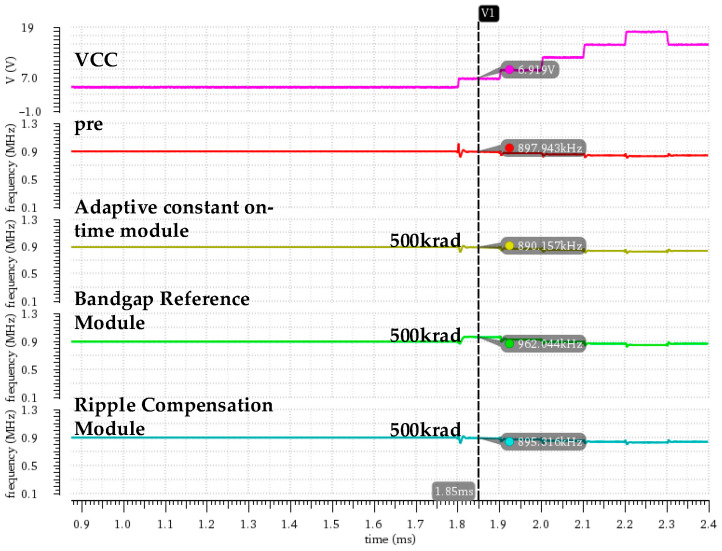
Variations in switching frequency after introducing the total-dose fault injection model into each key module.

Figure 11 shows variations in the switching frequency for the DCAP power chip after considering the total ionizing dose effect for each key module. The simulation results indicate that the different modules exhibit significant differences in their effects on switching frequency. The detailed simulation data are shown in Table 4, from which we can determine that, under the total-dose radiation, the bandgap reference module exhibits the most important effect on the switching frequency. At an input voltage of 7 V, the switching frequency increases from 897.1 kHz to 962.3 kHz and the frequency fluctuation range expands from 44.2 kHz to 109.6 kHz after irradiating the bandgap reference module, indicting a significant degradation of the constant frequency characteristics.

For the adaptive constant on-time module, the switching frequency is slightly decreased and the variation range is controlled within 10 kHz after injecting the total-dose radiation model. In addition, the constant frequency characteristics remain basically stable, indicating that the adaptive constant on-time module still has a strong frequency regulation capability under the total-dose environment. The ripple compensation module exhibits the smallest effect on switching frequency after the total-dose radiation, with a frequency change amplitude of less than 5 kHz and excellent constant frequency characteristics.

In conclusion, the above research reveals that the bandgap reference circuit is the most sensitive module in terms of affecting output accuracy and switching frequency, while the adaptive constant on-time module and ripple compensation module both exhibit better immunity and stability under the total ionizing dose effect.

### 3.3. Bandgap Reference Module

**Figure 12:**
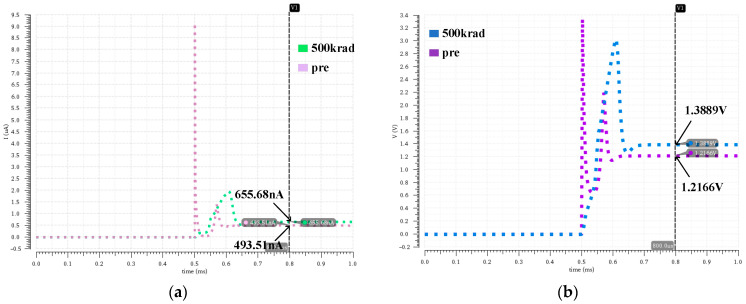
Variations in (**a**) bias current and (**b**) bias voltage after total-dose radiation.

Based on sensitivity analysis of each key module in the DCAP power chip, we found that output accuracy and switching frequency exhibited the most severe degradation when irradiating the bandgap reference module. Therefore, we mainly focused on the impact of the total ionizing dose effect on the bandgap reference module. To achieve this goal, we carried out a transient simulation to observe variations in bias current and bias voltage during the power-up process of the bandgap reference module, with a process condition of TT and a simulation time of 0–1 ms. Figure 12 illustrates the simulation results. These show that the bias current increases from 493.51 nA to 655.68 nA under an irradiation dose of 500 krad, and current remains constant after stabilization, while circuit stabilization time is significantly extended. Furthermore, we can also determine that the reference voltage is positively correlated with the bias current. The rise in the bias current causes the current flowing through the resistor to increase, thereby resulting in an increase in the reference voltage.

**Figure 13:**
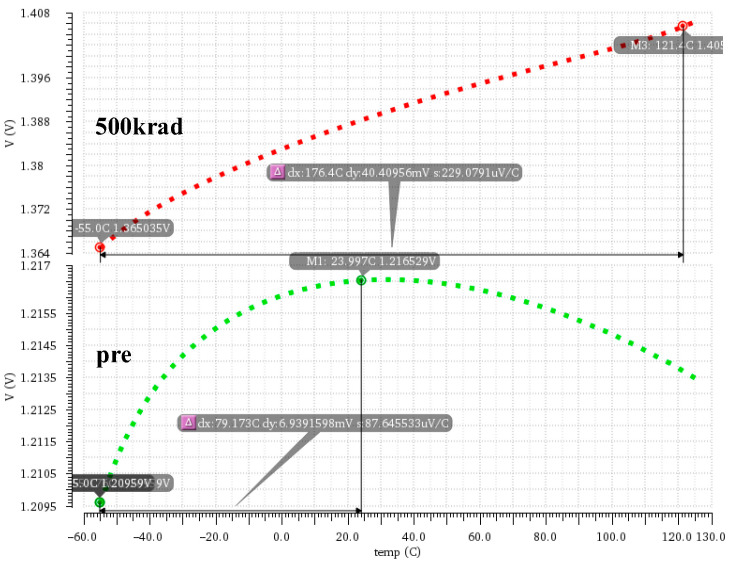
Reference voltage as a function of temperature before and after total-dose radiation.

To evaluate the temperature characteristics of the bandgap reference module under the total ionizing dose effect, we set the process condition as TT with a 3.6 V supply voltage across a temperature range from −55 °C to 125 °C. Figure 13 illustrates the temperature characteristic curves of the reference voltage before and after radiation at an irradiation dose of 500 krad. As we can see, before radiation, the reference voltage reaches the zero-temperature coefficient point near room temperature, with an output voltage of about 1.21 V. Meanwhile, across the range of temperatures, the maximum difference in the reference voltage is 6.93 mV, with a corresponding temperature drift coefficient of 72.3 ppm/°C. However, after radiation, the temperature drift is significantly aggravated, with the maximum voltage difference increasing to 40.41 mV and the entire temperature drift coefficient rising to 163.36 ppm/°C. This indicates a significant degradation of the bandgap reference module’s temperature stability under the total-dose radiation environment [28,29].

## 4. Conclusions

In this paper, simulation and analysis of the total ionizing dose effect are carried out for a DCAP power chip under the 0.18 μm BCD process. We establish a fault injection model of the MOS device and apply it to circuit-level simulation to research the responses of the entire system and key modules under total-dose radiation. The simulation results show that, for the DCAP power chip, the output accuracy is degraded to 24.92%, the switching frequency is increased, the constant frequency characteristics are deteriorated, and the frequency fluctuation range is increased from ±7.4% to ±13.1% under an irradiation dose of 500 krad. Further research reveals that the bandgap reference circuit is the module most sensitive to the total ionizing dose effect, with output accuracy being degraded to 13.85% when the fault injection is performed only for the bandgap reference module. In contrast, the adaptive constant on-time module and ripple compensation module exhibit smaller influences on the performance and stability of the DCAP power chip under the total-dose radiation. The research in this paper indicates that we should focus on the bandgap reference module for total-dose radiation hardening designs for the DCAP power chip. In future work, we will attempt to evaluate the effectiveness of potential hardening techniques that could be adopted for the DCAP power chip, such as using a gate-all-around NMOS structure, adding p+ protection rings and introducing an ultra-thin buried layer.

## Figures and Tables

**Table 1:** Output voltage ripple amplitude and accuracy before and after total-dose radiation as a function of input voltage at an irradiation dose of 500 krad.

$V_{CC}$/V	$V_{out}$/V (Pre-Irradiation)	$V_{out}$/V (500 krad)	Ripple Amplitude/mV (Pre-Irradiation)	Ripple Amplitude/mV (500 krad)	Output Accuracy (Pre-Irradiation)	Output Accuracy (500 krad)
5	1.7974	2.1632	±2.996	±2.668	−0.144%	+20.18%
7	1.7986	2.1938	±4.034	±3.666	−0.078%	+21.88%
9	1.8004	2.2214	±4.750	±4.595	+0.022%	+23.41%
12	1.8014	2.2438	±5.456	±5.599	+0.078%	+24.66%
15	1.8021	2.2486	±5.975	±6.345	+0.117%	+24.92%
18	1.8020	2.2426	±6.331	±6.906	+0.111%	+24.59%

**Table 2:** Switching frequency as a function of input voltage before and after total-dose radiation.

$V_{CC}$/V	$f_{SW}$/kHz (Pre-Irradiation)	$f_{SW}$/kHz (500 krad)
5	902.6	968.6
7	896.7	1027.3
9	877.7	990.1
12	858.9	947.3
15	847.1	916.3
18	835.3	892.3

**Table 3:** Output voltage and accuracy as a function of input voltage under an irradiation dose of 500 krad for each key module.

$V_{CC}$/V	$V_{out}$/V (Bandgap Reference Module)	$V_{out}$/V (Adaptive Constant On-Time Module)	$V_{out}$/V (Ripple Compensation Module)	Output Accuracy (Bandgap Reference Module)	Output Accuracy (Adaptive Constant On-Time Module)	Output Accuracy (Ripple Compensation Module)
5	2.0373	1.7974	1.7967	+13.18%	−0.144%	−0.183%
7	2.0458	1.7987	1.7998	+13.66%	−0.072%	−0.011%
9	2.0465	1.8005	1.8022	+13.69%	+0.028%	+0.122%
12	2.0478	1.8014	1.8039	+13.77%	+0.078%	+0.217%
15	2.0487	1.8019	1.8048	+13.82%	+0.106%	+0.267%
18	2.0493	1.8023	1.8054	+13.85%	+0.128%	+0.300%

**Table 4:** Switching frequency as a function of input voltage under an irradiation dose of 500 krad for each key module.

$V_{CC}$/V	$f_{SW}$/kHz (Pre-Irradiation)	$f_{SW}$/kHz (Bandgap Reference Module)	$f_{SW}$/kHz (Adaptive Constant On-Time Module)	$f_{SW}$/kHz (Ripple Compensation Module)
5	902.7	900.6	895.7	900.5
7	897.1	962.3	889.7	895.8
9	880.1	930.4	872.2	876.3
12	858.5	895.7	853.7	858.1
15	847.2	871.5	840.8	844.2
18	834.9	852.7	830.8	834.5

## Data Availability

The original contributions presented in the study are included in the article, further inquiries can be directed to the corresponding author.

## Abbreviations

The following abbreviations are used in this manuscript:

BCD	Bipolar-CMOS-DMOS
DCAP	Direct connection to the output CAPacitor
NASA	National Aeronautics and Space Administration
NMOS	N-Metal-Oxide-Semiconductor
TID	Total Ionizing Dose
TCAD	Technology Computer-Aided Design
SPICE	Simulation Program with Integrated Circuit Emphasis
STI	Shallow Trench Isolation
TT	Typical Typical
